# The Behaviour of 5-Hydroxymethylcytosine in Bisulfite Sequencing

**DOI:** 10.1371/journal.pone.0008888

**Published:** 2010-01-26

**Authors:** Yun Huang, William A. Pastor, Yinghua Shen, Mamta Tahiliani, David R. Liu, Anjana Rao

**Affiliations:** 1 Department of Pathology, Harvard Medical School and Immune Disease Institute, Boston, Massachusetts, United States of America; 2 Department of Chemistry and Chemical Biology and the Howard Hughes Medical Institute, Harvard University, Cambridge, Massachusetts, United States of America; Johns Hopkins School of Medicine, United States of America

## Abstract

**Background:**

We recently showed that enzymes of the TET family convert 5-mC to 5-hydroxymethylcytosine (5-hmC) in DNA. 5-hmC is present at high levels in embryonic stem cells and Purkinje neurons. The methylation status of cytosines is typically assessed by reaction with sodium bisulfite followed by PCR amplification. Reaction with sodium bisulfite promotes cytosine deamination, whereas 5-methylcytosine (5-mC) reacts poorly with bisulfite and is resistant to deamination. Since 5-hmC reacts with bisulfite to yield cytosine 5-methylenesulfonate (CMS), we asked how DNA containing 5-hmC behaves in bisulfite sequencing.

**Methodology/Principal Findings:**

We used synthetic oligonucleotides with different distributions of cytosine as templates for generation of DNAs containing C, 5-mC and 5-hmC. The resulting DNAs were subjected in parallel to bisulfite treatment, followed by exposure to conditions promoting cytosine deamination. The extent of conversion of 5-hmC to CMS was estimated to be 99.7%. Sequencing of PCR products showed that neither 5-mC nor 5-hmC undergo C-to-T transitions after bisulfite treatment, confirming that these two modified cytosine species are indistinguishable by the bisulfite technique. DNA in which CMS constituted a large fraction of all bases (28/201) was much less efficiently amplified than DNA in which those bases were 5-mC or uracil (the latter produced by cytosine deamination). Using a series of primer extension experiments, we traced the inefficient amplification of CMS-containing DNA to stalling of Taq polymerase at sites of CMS modification, especially when two CMS bases were either adjacent to one another or separated by 1–2 nucleotides.

**Conclusions:**

We have confirmed that the widely used bisulfite sequencing technique does not distinguish between 5-mC and 5-hmC. Moreover, we show that CMS, the product of bisulfite conversion of 5-hmC, tends to stall DNA polymerases during PCR, suggesting that densely hydroxymethylated regions of DNA may be underrepresented in quantitative methylation analyses.

## Introduction

DNA methylation and demethylation occur dynamically during early embryogenesis and play a crucial role in mammalian development [1]–[5]. Changes in DNA methylation status are associated with X-inactivation, imprinting, and the development of primordial germ cells [2]–[5]; moreover, DNA methylation is highly aberrant in cancer [6]–[9]. There is also a correspondence between DNA methylation status and gene expression: the promoters of silenced genes tend to be heavily methylated whereas the promoters of active genes tend to be hypomethylated [3]–[5]. 5-methyl cytosine (5-mC), the primary methylated base in DNA, constitutes only ∼1% of all DNA bases [2]–[5]. In somatic cells, 5-mC is found almost exclusively in the context of paired symmetrical methylation of the dinucleotide CpG [10], [11] whereas in embryonic stem (ES) cells, a substantial amount of 5-mC is also observed in non-CpG contexts [12], [13]. The majority of methylated CpGs are located in repetitive DNA elements, suggesting that cytosine methylation evolved as a defense against transposons and other parasitic elements in DNA [5].

We and others have shown that the modified base, 5-hydroxymethylcytosine (5-hmC), is present in mammalian DNA; specifically, 5-hmC constitutes ∼5% of all cytosine species present at CpGs in MspI and Taq^α^I sites in ES cell DNA, and ∼20% of all cytosine species present at CpG's in cerebellar Purkinje cell DNA [14], [15]. Since ES cells are highly proliferative while Purkinje cells are post-mitotic, the biological functions of 5-hmC may differ depending on cell type. There are several possible scenarios, not mutually exclusive. (i) Conversion of 5-mC to 5-hmC could result in the displacement of 5-methylcytosine-binding proteins (MBPs) from methylated DNA; indeed at least one MBP, MeCP2, does not bind DNA containing 5-hmC [16] ; (ii) 5-hmC may promote DNA demethylation. Replacement of 5-mC with 5-hmC may interfere with maintenance methylation catalysed by DNMT1 during cell division [17], resulting in ”passive“ DNA demethylation; alternatively, 5-hmC may be recognized as an aberrant base by DNA repair mechanisms that replace 5-hmC with cytosine, in a process equivalent to ”active“ (replication-independent) demethylation; (iii) 5-hmC may be recognized by dedicated binding proteins that recruit specialized chromatin-modifying partners, thus altering chromatin structure and DNA methylation status.

The TET proteins TET1, TET2 and TET3 are 2-oxoglutarate (2OG)- and Fe(II)-dependent oxygenases that catalyse the conversion of 5-mC to 5-hmC in DNA [14]. TET1 and TET2 are both implicated in human leukemia, again arguing for the physiological importance of 5-hmC. TET1 is an MLL fusion partner and thus a likely oncogene: t(10;11)(q22;q23) translocations which fuse the N-terminal region of the histone-3 lysine-4 (H3K4) methyltransferase MLL with the catalytic domain of TET1 have been found in several cases of acute myeloid and lymphocytic leukemias (AML, ALL) [18]–[21]. Conversely, a tumour suppressor function seems likely for TET2, based on many reports of homozygous null mutations and chromosomal deletions involving the *TET2* locus in myelodysplastic syndromes (MDS), myeloproliferative disorders (MPD) and frank myeloid malignancies ([22]–[27] ; reviewed in [28], [29]). 5-hmC may also be generated by DNMT1-mediated oxidation of cytosine with formaldehyde, although this has yet to be demonstrated to occur under physiologically relevant conditions [30] .

**Figure 1:**
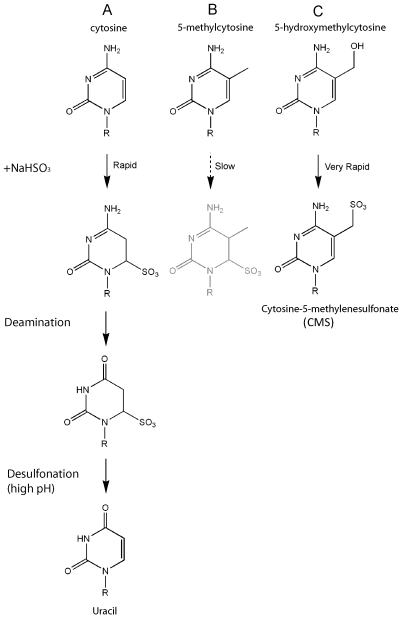
Reaction of sodium bisulfite with C, 5-mC and 5-hmC. (A) Bisulfite-mediated deamination of cytosine. HSO_3_^−^ reversibly and quickly adds across the 5,6 double bond of cytosine, promoting deamination at position 4 and conversion to 6-sulfonyluracil. 6-sulfonyluracil is stable under neutral conditions, but is easily desulfonated to uracil (U) at higher pH. (B) 5-methylcytosine is deaminated to thymine by bisulfite conversion, but the rate is approximately two orders of magnitude slower than that of cytosine. (C) Bisulfite quickly converts 5-hydroxymethylcytosine to form cytosine-5-methylenesulfonate (CMS). This adduct does not readily undergo deamination [45].

Taken together, these studies indicate that 5-hmC may have important roles distinct from 5-mC. It is thus critical to understand how 5-hmC behaves in techniques geared at mapping 5-mC. The genomic location of 5-mC has been mapped in several ways. (i) The 5-mC-binding domains of MBPs such as MeCP2, as well as antibodies against 5-mC, have been used to precipitate methylated DNA [31]–[36]. These reagents will not precipitate 5-hmC, as neither the commonly used 5-mC antibody nor the MeCP2 MBD domain bind 5-hmC effectively [14], [16]. (ii) Methylation-sensitive enzymes such as HpaII or McrBC do not reliably distinguish 5-mC and 5-hmC [37], [38]. (iii) For mapping 5-mC at single-base resolution, either at specific loci or at the genome-wide level, the most widely-used method is treatment with sodium bisulfite followed by PCR amplification and sequencing [32], [39], [40]. The bisulfite technique relies on the fact that reaction with sodium bisulfite promotes deamination of unmethylated C to yield U, which is read as T after PCR amplification (**Figure 1A**), whereas 5-mC reacts poorly with bisulfite and therefore is deaminated much more slowly than C (**Figure 1B**). As a result, unmethylated C is read as T in subsequent PCR reactions, whereas 5-mC is read as C [39], [40].

In this study, we examined the behaviour of 5-hmC-containing DNA in bisulfite analysis. Sodium bisulfite reacts with 5-hmC to yield a distinct adduct, cytosine 5-methylenesulfonate (CMS) [41] (**Figure 1C**). We confirmed, as shown previously [41] , that 5-hmC is not deaminated after bisulfite treatment, implying that a proportion of genomic loci identified as methylated may actually be hydroxymethylated. We also show that the CMS adduct tends to stall DNA polymerases during PCR, especially if these modified bases are adjacent to one another or spaced 1–2 nucleotides apart; this result suggests that genomic regions containing closely-spaced 5-hmC could be missed or underrepresented in quantitative methylation analyses.

## Results

### Sodium Bisulfite Treatment Does Not Distinguish between 5-mC and 5-hmC

**Figure 2:**
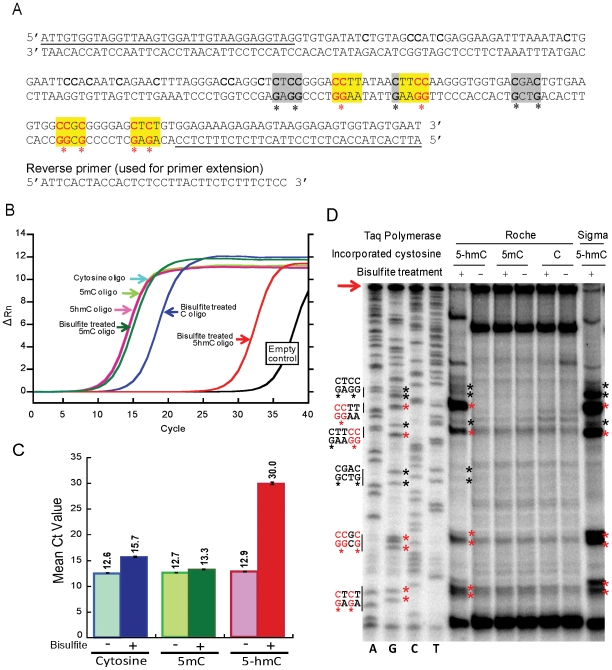
The bisulfite adduct of 5-hmC hinders PCR amplification. (A) Sequence of oligonucleotide containing multiple cytosines (used in **Figures 2B-D**). The yellow highlighted sequences and the red asterisks indicate the sequences and cytosine (putative CMS) residues at which DNA polymerases tended to stall when bisulfite-treated 5-hmC-containing DNA was used as template, whereas the grey highlighted sequences and black asterisks indicate the sequences and cytosine (putative CMS) residues that cause weak or no stalling by the DNA polymerases (see **Figure 2D**). Cytosines in the first 106 bases of the oligonucleotide are difficult to distinguish via Sanger sequencing and thus are not annotated with regard to stalling. The underlined sequences correspond to the forward and reverse PCR primers used for PCR amplification. (B) Real-time PCR amplification curve of an oligonucleotide containing C, 5-mC or 5-hmC before and after bisulfite treatment. The sequence of the oligonucleotide is shown in **Figure 2A**. The small lag observed for the bisulfite-treated cytosine oligonucleotide is due in part to the fact that after conversion of cytosine to uracil, this oligonucleotide can only be amplified from one of the two strands. (C) Quantification of Ct value from experiments performed as in **Figure 2B**. The mean and standard deviation of three experiments is shown. (D) Primer extension assays for DNA containing different cytosine species, shown beside a Sanger sequencing ladder. Ladders of incomplete extension products were only observed in the 5-hmC-containing DNA after bisulfite treatment, at positions corresponding to G in the Sanger sequencing ladder (*left lanes*). Red asterisks: positions with the most significant stalling; black asterisks: positions with weak stalling or no stalling. The corresponding sequences are indicated on the left (please compare with **Figure 2A**). The extension reaction performed with bisulfite-treated 5-hmC-containing DNA yielded less full-length product (arrow).

To test how 5-hmC affects bisulfite sequencing, we generated DNA templates containing C, 5-mC or 5-hmC as their sole cytosine species. To do this, we amplified a 201 bp oligonucleotide (**Figure 2A**) by PCR, using the nucleoside triphosphates dATP, dGTP, dTTP and either dCTP or its 5-mC or 5-hmC derivatives. The top strand of this oligonucleotide (201-bp) contains 28 randomly distributed cytosines. Cytosine was avoided in the primer-annealing region to ensure efficient annealing after bisulfite treatment. The PCR products were treated with bisulfite and exposed to conditions promoting deamination and desulfonation.

**Figure 3:**
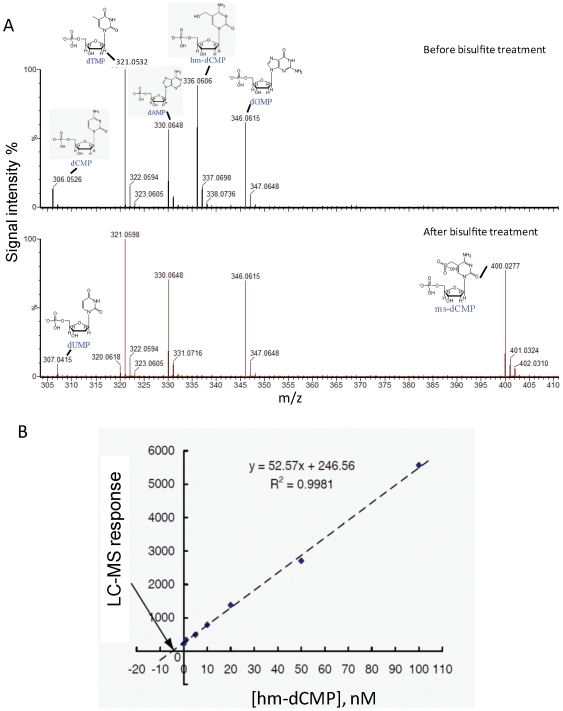
LC-MS analysis of conversion efficiency of 5-hmC to CMS. (A) MS analysis of the nuclease P1 digestion products of the oligonucleotides used in **Figure 2A**, before (upper panel) and after (lower panel) bisulfite treatment. (B) To determine the conversion efficiency of 5-hmC to CMS in the oligonucleotide shown in **Figure 2A**, a standard curve was used to determine the unknown quantity of hmdCMP in the sample before and after treatment with sodium bisulfite (see text for details). The absolute value of the intercept of the best-fit line with the X-axis gives the concentration of hmdCMP remaining in the sample after bisulfite treatment as 4.69 nM. Given that the hmdCMP concentration before bisulfite treatment was 1.5 µM, this corresponds to a conversion efficiency as high as 99.7%.

We first measured the efficiency of C>T conversion after bisulfite treatment (**Figure 3**). The 201 bp oligonucleotide, before and after bisulfite treatment (**Figure 3A**, upper and lower panels respectively), was digested with nuclease P1 and subjected to LC-MS analysis. Observed m/z values and corresponding dNMP structure are indicated (mass accuracy is within 0.002 Da). After bisulfite treatment, hm-dCMP (**Figure 3A**, upper panel, peak at 336.0606 Da) is not detectable because it is converted to ms-dCMP (lower panel, peak at 400.0277 Da). Similarly, the cytosines in the primers (upper panel, peak at 306.0526 Da) undergo conversion to uracil, resulting in the peak corresponding to dUMP (lower panel, peak at 307.0415 Da).

To determine the conversion efficiency, a standard curve was generated (**Figure 3B**). Seven aliquots were taken from the reaction mixture after treatment with sodium bisulfite, and a known amount of authentic hmdCMP was added to each aliquot, corresponding to a final concentration of 0, 1 nM, 5 nM, 10 nM, 20 nM, 50 nM, or 100 nM added hmdCMP. Each of the resulting samples was analyzed by LC/MS: 10 µL of each sample was injected, and duplicate LC/MS analyses were performed for each sample. The average ion abundance of [M–H] m/z = 336.06 for each sample was plotted as a linear function of the concentration of authentic hmdCMP added. The absolute value of the intercept of the best-fit line with the X-axis provides the concentration of hmdCMP remaining in the original sample after bisulfite treatment (calculated to be 4.69 nM). Since the hmdCMP concentration before bisulfite treatment was 1.5 µM, this level of remaining hmdCMP corresponds to a conversion efficiency of 99.7%.

**Figure 4:**
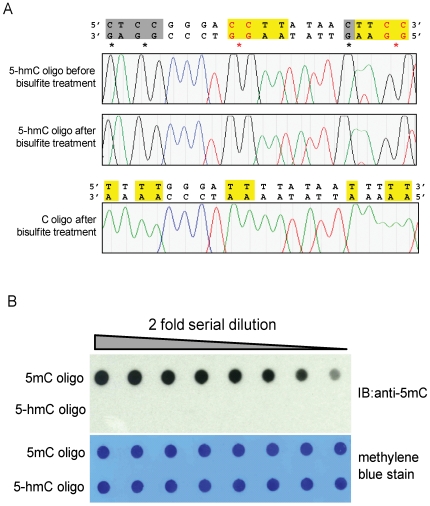
5-hmC did not undergo C->T transitions after bisulfite treatment and 5-mC antibody cannot recognize 5-hmC in DNA. (A) Shown are sequencing traces of 5-hmC-containing oligonucleotide (**Figure 2A**) before and after bisulfite treatment (*top and middle panels*). The control C-containing oligonucleotide shows complete conversion of all C's in the top strand (highlighted sequences) to T's (*lower panel*). (B) Dot-blot assay of monoclonal anti-5-mC antibody detection of oligonucleotide (**Figure 2A**) containing 5-mC or 5-hmC (**Figure 2A**). Recognition on DNA by the anti-5-mC antibody is shown in the top panel, loading control is shown by the methylene blue stain in the bottom panel. The anti-5-mC antibody only recognizes the 5-mC oligonucleotide but not the 5-hmC oligonucleotide.

We then sequenced the amplified DNA oligonucleotides before and after bisulfite treatment. Sequencing confirmed that all cytosines in the oligonucleotide were converted to thymines after bisulfite treatment; a representative sequence is shown (**Figure 4A**, lower panel). In contrast, bisulfite-treated 5-hmC did not undergo C->T transitions, as expected from its chemical and base pairing properties [41] (**Figure 4A**, upper panels). Since 5-mC also does not undergo conversion under these conditions [41], our results indicate that the widely-used bisulfite sequencing technique fails to distinguish between 5-mC and 5-hmC. To test whether commercially available anti-5mC antibodies recognize 5-hmC, equivalent amounts of 5-mC- or 5-hmC- containing oligonucleotide were spotted on a nitrocellulose membrane (**Figure 4B**), incubated with anti 5-mC antibody, and visualized via chemiluminescence. 5-hmC was not recognized by the anti-5mC antibody (**Figure 4B**). This indicates that sites of hydroxymethylation would likely appear methylated by bisulfite sequencing but unmethylated by detection techniques that rely on antibody [14], [16].

### The Bisulfite Adduct of 5-hmC Hinders PCR Amplification

We next asked if the presence of the bulky CMS adduct might hinder PCR amplification. The PCR-amplified oligonucleotides containing cytosine, 5-mC or 5-hmC were treated with bisulfite and amplified with the primers shown in **Figure 2A**. The amplification efficiencies were measured by real-time PCR. Under these conditions, 5-hmC-containing DNA was very inefficiently amplified compared to C- and 5-mC-containing DNA (**Figures 2B, C**).

To determine where the block in PCR amplification occurred, we performed primer extension assays using two commercial sources of Taq DNA polymerase. A ladder of incomplete extension products was seen only with bisulfite-treated, 5-hmC-containing DNA **(** **Figure 2D** **)**. The most significant stalling (marked with red asterisks in **Figures 2A, D**) occurred at positions across from a CTC sequence close to the end of the reverse primer, and a CCGC sequence and several CC sequences further away. Cytosine residues where stalling was weak or did not occur are marked with black asterisks (**Figures 2A, D**). The results suggest that CMS stalls but does not completely block Taq polymerase, and that the stalling is particularly pronounced when two CMS nucleotides are adjacent to one other or separated by a single nucleotide as in the CTC sequence.

### The Bisulfite Adduct of 5-hmC Stalls Taq Polymerase at CpG Dinucleotides

**Figure 5:**
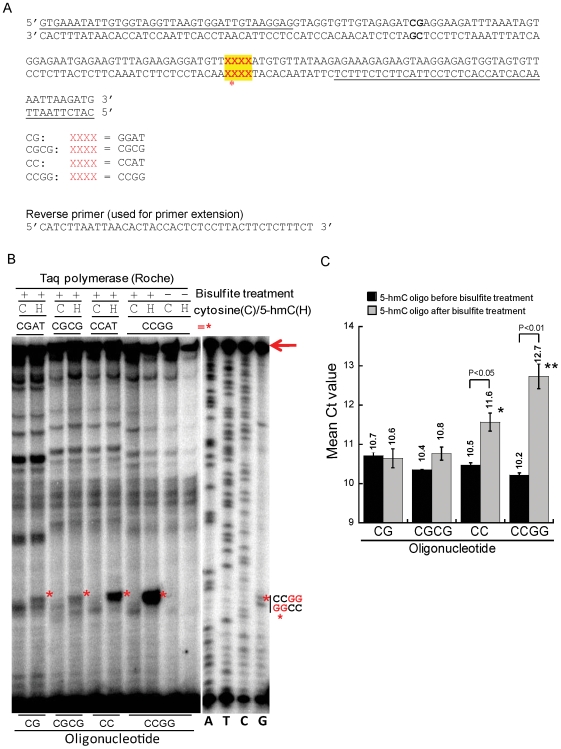
The bisulfite adduct of 5-hmC stalls Taq polymerase at CpG dinucleotides. (A) Sequences of a set of five 158 bp oligonucleotides used in this assay. At the position marked XXXX (red font with yellow highlight), the CG oligonucleotide contains the sequence CGAT, the CGCG oligonucleotide contains two tandem CGs, and the CC and CCGG oligonucleotides contain CCAT and CCGG sequences respectively. The underlined sequences correspond to the forward and reverse PCR primers used for PCR amplification. (B) Primer extension assays of oligonucleotides shown in **Figure 5A**. The bands corresponding to stalled PCR reactions (red asterisks, see XXXX in **Figure 5A**) were most prominent in 5-hmC-containing CC and CCGG oligonucleotides after bisulfite treatment, and were observed, though less obvious, in the CG and CGCG oligonucleotides. Full length product is indicated by an arrow. *Right lanes*, the Sanger sequencing was performed using the CCGG oligonucleotide as a template. (C) Quantification of Ct value of real-time PCR from experiments performed on the substrates used in **Figure 5A**. The mean and standard deviation of three experiments is shown.

In mammalian DNA, 5-mC (and presumably its hydroxylated derivative, 5-hmC) are found almost exclusively in the context of the dinucleotide CpG [10], [11]; however, DNA from embryonic stem cells contains 5-mC in non-CpG contexts [12], [13]. To evaluate the degree to which CMS would stall Taq polymerase in this physiological context, we synthesized a set of 158 bp oligonucleotides in which the top strand contained a variable sequence that was one of the following: CGAT, CCAT, CGCG, or CCGG (indicated by XXXX in **Figure 5A**). After bisulfite treatment, the most significant stalling was observed at the tandem CC sequences in the CC and CCGG oligonucleotides (red asterisks in **Figure 5B**, *lanes 6, 8*). Bisulfite-dependent stalling was also observed to a lesser degree at the same position in the CG and CGCG oligonucleotides (red asterisks in **Figure 5B**, *lanes 2, 4*).

Consistent with these observations, the CG and CGCG oligonucleotides were efficiently amplified after bisulfite treatment, whereas oligonucleotides containing CC sequences showed a perceptible decrease in amplification efficiency (**Figure 5C**). Note that the PCR amplification is performed with standard nucleotides, yielding PCR products that contain A, C, G and T but no CMS. Therefore, the observed difference in Ct values between bisulfite-treated and untreated CC and CCGG oligonucleotides most likely arises from inefficient initial production of full-length PCR products; once generated, full-length PCR products will be amplified as efficiently as any other DNA.

To summarize, we have traced the inefficient amplification of CMS-containing DNA to the fact that CMS residues tend to stall Taq polymerases. The extent of stalling varies with DNA sequence and with the polymerase used, but is perceptible in the context of a single CMS-guanine dinucleotide and is pronounced in sequence contexts where CMS residues are adjacent or within 1–2 nucleotides of one another.

## Discussion

In summary, we have confirmed that 5-hmC does not undergo C-to-T transitions after bisulfite treatment, and thus cannot be distinguished from 5-mC by the bisulfite technique. In addition, we find that primer extension reactions conducted with bisulfite-treated DNA terminate disproportionately at sites of hydroxymethylation, bringing up the distinct possibility that bisulfite-based analyses of DNA methylation status could miss or underestimate the occurrence of DNA regions with dense hydroxymethylation.

We initiated these studies in light of the recent discovery, by us and others, that 5-hmC is a bona fide constituent of mammalian DNA, especially in ES cells and Purkinje neurons [14], [15]; and our own finding that the enzymes that catalyse the conversion of 5-mC to 5-hmC in DNA belong to the TET protein family [14]. There has been a recent proliferation of studies in which the DNA methylation status of plant and mammalian genomes has been mapped, most recently using whole-genome bisulfite sequencing alone [13], but more often through bisulfite sequencing of DNA enriched by MeDIP (immunoprecipitation of methylated DNA using either specific antibodies to 5-mC or the methyl-CpG-binding domains of MBPs such as MeCP2) [31]–[36] . Specifically, genome-wide maps of DNA methylation were recently achieved for *Arabidopsis thaliana* and for human ES cells by generating bisulphite sequencing libraries compatible with next-generation deep sequencing (BiS-seq) [13], [32].

Many of the mammalian mapping studies have been performed in embryonic stem cells, which are known to contain 5-hmC [14], or in cancer cell lines, in which DNA methylation is known to be aberrant [6]–[9]. We therefore tackled the question of how 5-hmC might be interpreted in the traditional bisulfite-based methods of DNA methylation analysis. We used two synthetic oligonucleotides with different distributions of cytosine species to compare the behaviour of C, 5-mC and 5-hmC in the bisulfite technique. The first of these oligonucleotides contained C, 5-mC or 5-hmC as their sole cytosine species in the top strand in both CpG and non-CpG contexts (28 cytosines/201 bases, or 14%). We first confirmed by mass spectrometry that >99% of 5-hmC was converted to the expected CMS adduct [41] upon reaction with bisulfite (**Figure 1**), and that CMS, like 5-mC, was resistant to deamination and therefore was read as C upon PCR amplification (**Figure 2**). We also found, however, that this oligonucleotide was very poorly amplified after PCR (**Figure 2A**), suggesting that the bulky CMS adduct generated by the reaction of bisulfite with 5-hmC (**Figure 1**) interfered with PCR amplification. We traced the problem to the fact that two different Taq polymerases, both constituents of commercial bisulfite kits, were stalled by CMS, especially in regions of dense hydroxymethylation where two CMS residues were adjacent or were separated by only one or two nucleotides.

Since much cytosine methylation in mammalian cells occurs in the context of CpG dinucleotides [11]–[13], we designed a second oligonucleotide that contained one CpG and an additional sequence of four bases that included CC, CG, CCGG or CGCG. Again, stalling was prominent at tandem CC sequences, which would be converted to tandem CMS sequences after bisulfite treatment (**Figure 5**). However, there was also clear bisulfite-dependent stalling at the CpG sequences in the CG and CGCG oligonucleotides. These findings imply that bisulfite sequencing data should be interpreted with caution, since loci containing dense regions of hydroxymethylated DNA might be incorrectly assumed to contain methylated CpGs, and might also be underrepresented in quantitative analyses of DNA methylation status.

Notably, genome-wide analyses of cytosine methylation in ES cells has shown that ∼25% of all 5-mC is in a non-CpG context; two C's that are immediately adjacent can both be methylated [13]. We have shown that the CC sequence context is particularly liable to stall Taq polymerases after 5-hmC>CMS conversion (**Figures 2D** **,** **5B**), therefore DNA regions that contain tandem 5-hmC's might be under-represented through inefficient PCR amplification after bisulfite treatment. At present it is difficult to test this possibility in mammalian genomic DNA: no 5-hydroxymethylated loci have been identified, and immunoprecipitation strategies to identify endogenous 5-hmC-containing loci in ES or Purkinje cell DNA have not yet been developed.

It may be possible in future to exploit our finding that primer extension reactions conducted with bisulfite-treated DNA terminate disproportionately at sites of hydroxymethylation. Primer extensions with appropriate polymerases could be performed, possibly under suboptimal extension conditions, and combined with ligation-mediated PCR to establish the genomic location of 5-hmC at single-base (“horizontal“ [39]) resolution.

It is unclear how CMS inhibits PCR. Rein *et al*. proposed that the bulky CMS adduct would block DNA polymerase by analogy to oxidative pyrimidine adducts such as thymine glycol [42], [43] or 6-sulfonyluracil [44]. However, CMS retains aromaticity, whereas it has been demonstrated that polymerases are disrupted by thymine glycol's loss of aromaticity and consequent adoption of a six-membered ring chair geometry [12]. Whatever the mechanism, the observation that 5-hmC can stall Taq polymerase after bisulfite reactions has important ramifications for our interpretation of previous DNA methylation analyses as discussed above.

## Materials and Methods

### Design of Minigenes for Generation of DNA Templates Containing C, 5-mC or 5-hmC

Minigenes used as templates to amplify C, 5-mC or 5-hmC containing oligonucleotides (**Figure 2** and **Figure 5**) were synthesized by Integrated DNA Technologies. DNA containing C, 5-mC or 5-hmC was amplified by PCR using 0.2 mM nucleoside triphosphates dATP, dGTP, dTTP with dCTP or its derivatives mdCTP (GE Healthcare) or hmdCTP (Bioline). PCR products were run on a 2% agarose gel to confirm correct length and further purified by a gel extraction kit (Qiagen).

### Bisulfite Treatment of Oligonucleotides

Bisulfite treatment and recovery of samples were carried out with the EpiTect Bisulfite kit (QIAGEN) by following the manufacturer's instructions. Briefly, 2 µg DNA in 20 µL volume was used for each reaction and mixed with 85 µL bisulfite mix and 35 µL DNA protect buffer. Bisulfite conversion was performed on a thermocycler as follows: 99°C for 5 min, 60°C for 25 min, 99°C for 5 min, 60°C for 85 min, 99°C for 5 min, 60°C for 175 min and 20°C indefinitely. The bisulfite-treated DNA was recovered by EpiTect spin column and subsequently sequenced to confirm the efficiency of bisulfite conversion.

### Liquid Chromatography/Mass Spectrometry Analysis

The bisulfite-treated oligonucleotide shown in **Figure 2** was precipitated with ethanol, digested by nuclease P1, lyophilized, and redissolved in water for liquid chromatography/mass spectrometry (LC/MS) analysis using an Acquity UPLC/Q-TOF Premier electrospray LC/ESI-MS system (Waters Corp., Milford, MA). Liquid chromatography was performed with a Waters HSS C18 column (1.0 mm i.d. ×50 mm, 1.8-um particles) using a linear gradient of 0% to 100% methanol in 0.1% aqueous ammonium formate, pH 6.0. The flow rate was 0.03 mL/min and the eluant was directly injected into the mass spectrometer. The data were analyzed using the Masslynx 4.1 software package (Waters).

### RealTime PCR of Oligonucleotides

RealTime PCR was performed on the StepONE plus real-time PCR system (Applied Biosystems) by using the FastStart Universal SYBR Green Master kit (Roche). 0.1 µg DNA template and 0.15 mM primers were used in each reaction in a final volume of 20 µL. The amplification reaction program was set as: 95°C for 10 min, 40 cycles of 95°C for 15 sec, 60°C for 1 min, and a melt curve analysis step at the end. Data were analyzed by StepONE plus real-time PCR software.

### Primer Extension Assay

Reverse primers (50 ng) were end labeled with T4 polynucleotide kinase (T4 PNK) (NEB) and 10 µCi of [γ-^32^P]-ATP (PerkinElmer) for 1 hr at 37°C, and then purified by Illustra MicroSpin G-25 column (GE Healthcare). For the primer extension, 2 ng template and 4 pmol γ^32^-P-labeled primers were used in a final volume of 20 µL. PCR reactions were set up according to the manufacturer's instructions using two commercial sources of Taq DNA polymerase (Roche and Sigma). For Roche Taq DNA polymerase, PCR conditions were: 95°C for 10 min, 30 cycles of 95°C for 15 sec, 60°C for 1 min. For Sigma TaqRED polymerase, PCR conditions were: 30 cycles of 94°C for 1 min, 55°C for 2 min and 72°C for 1 min. The primer extension products were mixed with 2X gel loading buffer II (Ambion), denatured at 95°C for 15 min and loaded on to a 12% denaturing polyacrylamide gel (7 M urea). Sanger sequencing was performed using Thermo Sequenase Dye Primer Manual Cycle Sequencing kit (USB). 2 ng template and 1 pmol [γ^32^-P]-labeled primer were used for Sanger sequencing. The results were visualized by autoradiography.

### Dot-Blot Assay

5-mC and 5-hmC oligonucleotides were generated as described above. 2 µg of DNA was denatured in 0.4 M NaOH, 10 mM EDTA at 95°C for 10 min, and then neutralized by adding an equal volume of cold 2 M ammonium acetate (pH 7.0). Next, 2-fold dilutions of denatured DNA samples were spotted on a nitrocellulose membrane in an assembled Bio-Dot apparatus (Bio-Rad). Vacuum was subsequently applied to filter through DNA samples. The blotted membrane was washed with 2x SSC buffer, air-dried and vacuum-baked at 80°C for 2 hrs. The membrane was then blocked with 5% non-fat milk and incubated with monoclonal 5-mC antibody (1∶1000) (Calbiochem). Binding of an HRP-conjugated secondary antibody (1∶12000) was visualized by enhanced chemiluminescence. To ensure equal spotting of total DNA on the membrane, the same blot was then stained with 0.02% methylene blue in 0.3 M sodium acetate (pH 5.2).
